# Exploring Bioequivalence of Dexketoprofen Trometamol Drug Products with the Gastrointestinal Simulator (GIS) and Precipitation Pathways Analyses

**DOI:** 10.3390/pharmaceutics11030122

**Published:** 2019-03-15

**Authors:** Marival Bermejo, Gislaine Kuminek, Jozef Al-Gousous, Alejandro Ruiz-Picazo, Yasuhiro Tsume, Alfredo Garcia-Arieta, Isabel González-Alvarez, Bart Hens, Deanna Mudie, Gregory E. Amidon, Nair Rodriguez-Hornedo, Gordon L. Amidon

**Affiliations:** 1 Department of Pharmaceutical Sciences, College of Pharmacy, University of Michigan, Ann Arbor, MI 48109, USA; gkuminek@umich.edu (G.K.); jalgouso@umich.edu (J.A.-G.); ytsume@umich.edu (Y.T.); barthens@umich.edu (B.H.); deanna.mudie@lonza.com (D.M.); geamidon@umich.edu (G.E.A.); nrh@umich.edu (N.R.-H.); glamidon@umich.edu (G.L.A.); 2 Department Engineering Pharmacy Section, Miguel Hernandez University, San Juan de Alicante, 03550 Alicante, Spain; alejandroruizpicazo@gmail.com (A.R.-P.); isabel.gonzalez@goumh.umh.es (I.G.-A.); 3 Department of Biopharmaceutics and Pharmaceutical Technology, Johannes Gutenberg Universität Mainz, D-55099 Mainz, Germany; 4 Merck and Co., Inc., 126 E Lincoln Ave, Rahway, NJ 07065, USA; 5 Service on Pharmacokinetics and Generic Medicines, Division of Pharmacology and Clinical Evaluation, Department of Human Use Medicines, Spanish Agency for Medicines and Health Care Products, 28022 Madrid, Spain; agarciaa@aemps.es; 6 Department of Pharmaceutical and Pharmacological Sciences, KU Leuven, Herestraat 49, 3000 Leuven, Belgium; 7 Drug Product Development and Innovation, Lonza Pharma and Biotech, Bend, OR 97703, USA

**Keywords:** gastrointestinal absorption, dexketoprofen, gastrointestinal simulator, microscopy imaging, liquid–liquid phase separation, oral absorption, in vitro dissolution

## Abstract

The present work aimed to explain the differences in oral performance in fasted humans who were categorized into groups based on the three different drug product formulations of dexketoprofen trometamol (DKT) salt—Using a combination of in vitro techniques and pharmacokinetic analysis. The non-bioequivalence (non-BE) tablet group achieved higher plasma C_max_ and area under the curve (AUC) than the reference and BE tablets groups, with only one difference in tablet composition, which was the presence of calcium monohydrogen phosphate, an alkalinizing excipient, in the tablet core of the non-BE formulation. Concentration profiles determined using a gastrointestinal simulator (GIS) apparatus designed with 0.01 N hydrochloric acid and 34 mM sodium chloride as the gastric medium and fasted state simulated intestinal fluids (FaSSIF-v1) as the intestinal medium showed a faster rate and a higher extent of dissolution of the non-BE product compared to the BE and reference products. These in vitro profiles mirrored the fraction doses absorbed in vivo obtained from deconvoluted plasma concentration–time profiles. However, when sodium chloride was not included in the gastric medium and phosphate buffer without bile salts and phospholipids were used as the intestinal medium, the three products exhibited nearly identical concentration profiles. Microscopic examination of DKT salt dissolution in the gastric medium containing sodium chloride identified that when calcium phosphate was present, the DKT dissolved without conversion to the less soluble free acid, which was consistent with the higher drug exposure of the non-BE formulation. In the absence of calcium phosphate, however, dexketoprofen trometamol salt dissolution began with a nano-phase formation that grew to a liquid–liquid phase separation (LLPS) and formed the less soluble free acid crystals. This phenomenon was dependent on the salt/excipient concentrations and the presence of free acid crystals in the salt phase. This work demonstrated the importance of excipients and purity of salt phase on the evolution and rate of salt disproportionation pathways. Moreover, the presented data clearly showed the usefulness of the GIS apparatus as a discriminating tool that could highlight the differences in formulation behavior when utilizing physiologically-relevant media and experimental conditions in combination with microscopy imaging.

## 1. Introduction

The development of generic oral drug products containing dexketoprofen trometamol (DKT, weak acid salt, Biopharmaceutics Classification System (BCS) class 1 drug) is challenging as the reference product does not dissolve rapidly. Since the dissolution of the reference product is not complete (<85%) in 30 min in the paddle apparatus at 50 rotations per minute (rpm) in any of the Biopharmaceutics Classification System’s (BCS) buffer media, a biowaiver approach is currently not permitted [1,2].

In Spain, three out of four formulations of DKT tablets failed the first in vivo bioequivalence (BE) study [3]. These products were previously tested with the European Medicines Agency (EMA) dissolution method requested for biowaiver applications, i.e., performing dissolution tests in USP-2 apparatus at 50 rpm with different buffers at pH 1.2, 4.5, and 6.8. Garcia-Arieta and co-workers showed the relevance of the agitation rate (50 rpm versus 75 rpm) on the dissolution profile outcomes [3]. Dissolution profiles of one DKT product using USP apparatus 2 (pH 1.2, 4.5, and 6.8) exhibited profiles (*f*2 < 50) that were not similar to in vivo BE. Another product exhibited in vitro BE (*f*2 > 50) but failed the in vivo BE study. Therefore, the USP apparatus 2 did not reflect the in vivo BE outcome.

The aim of this work was to determine the reasons for the differences in dissolution behavior between bioequivalent (BE) and non-bioequivalent (non-BE) DKT products. First, a physiologically-relevant, multi-compartmental dissolution apparatus, the gastrointestinal simulator (GIS), was evaluated to ascertain whether it could reflect the in vivo BE outcomes. Both the DKT products as well as the reference product were studied in the GIS. In the second step, salt to free acid precipitation pathways during dissolution of DKT were examined by inverted microscopy to identify the factors that influenced drug precipitation.

## 2. Materials and Methods

### 2.1. Chemicals

Three different formulations were tested in the GIS and USP-2 apparatus: the reference Spanish marketed product (Enantyum^®^, Laboratorios Menarini S.A., Barcelona, Spain) and two generic drug products. Acetonitrile was obtained from VWR International (West Chester, PA, USA). Methanol (MeOH), HCl, and trifluoroacetic acid (TFA) were purchased from Fisher Scientific (Pittsburgh, PA, USA). NaOH, NaCl, and NaH_2_PO_4_.H_2_O were received from Sigma-Aldrich (St. Louis, MO, USA). Purified water (i.e., filtrated and deionized) was used in the analysis methods and in dissolution studies to prepare the dissolution media (Millipore, Billerica, MA, USA). Simulated intestinal fluid (SIF) powder was obtained from Biorelevant (Croydon, UK).

Table 1 represents the qualitative composition for each formulation in terms of excipients and coating material.

The main difference between both test products is that there is calcium phosphate in the tablet core of the non-BE product.

### 2.2. Design of the In Vitro Dissolution Studies Performed with the GIS

**Figure 1:**
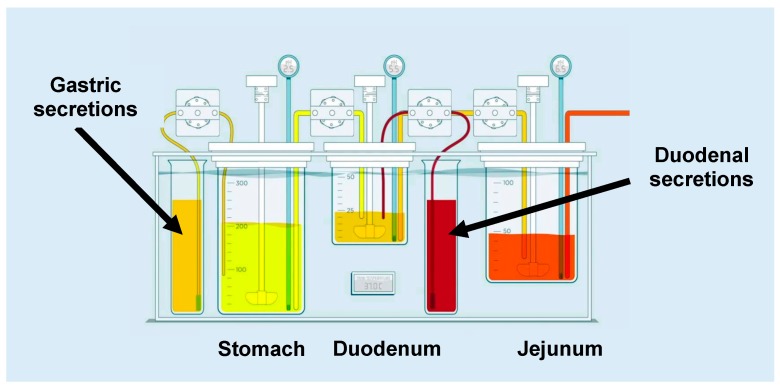
Setup and design of the gastrointestinal simulator (GIS) that was applied to test the different formulations of dexketoprofen trometamol (DKT) in fasted state conditions. Figure adopted from Hens and Bermejo et al. [4] with permission. Copyright Elsevier 2018.

The GIS is a three-compartmental dissolution device, which consists of (i) a gastric chamber (GIS_stomach_), (ii) a duodenal chamber (GIS_duodenum_), and (iii) a jejunal chamber (GIS_jejunum_). The design of the GIS is depicted in Figure 1.

The different dissolution protocols that were applied to test the different formulations in the multicompartmental GIS device are shown in Table 2 and Table 3. Table 2 represents the dissolution experiments that were performed in the absence of NaCl (i.e., Protocol 1). The gastric chamber contained simulated gastric fluid (SGF) and the duodenal compartment contained phosphate buffer, pH 6.8 (50 mM). We will refer to this test condition as the standard dissolution “Protocol 1” throughout the manuscript. To explore the impact of endogenous constituents present in the stomach (i.e., NaCl) and in the small intestine (i.e., bile salts and phospholipids), Protocol 2 was developed. In that case, the impact of NaCl on the conversion from salt to free acid could be investigated in the gastric compartment (i.e., acidic pH), and how the created solution concentrations will further behave in the duodenal compartment in a more biorelevant setting. Table 3 represents a higher level of biorelevant dissolution testing by using SGF in the gastric compartment in the presence of NaCl. The duodenal compartment contains fasted state simulated intestinal fluid (FaSSIF-v1). We will refer to this test condition as the standard dissolution “Protocol 2” throughout the manuscript.

The above-mentioned formulations were introduced into the GIS_stomach_ at the start of the experiment. Gastric emptying was set to a first-order kinetic process with a rate corresponding to a gastric half-life of 13 min, in accordance with the reported half-life in humans for liquids, ranging from 4 to 13 min [5]. Duodenal volume was kept constant at 50 mL by balancing the input (i.e., gastric emptying and duodenal secretion) with the output flow. The jejunal compartment was empty at the beginning of the experiment. Fluid from GIS_stomach_ was transferred to the GIS_duodenum_ and then to the GIS_jejunum_ with the aid of two Ismatec REGLO peristaltic pumps (IDEX Health and Science, Glattbrugg, Switzerland). Same pumps were used for the gastric and duodenal secretion fluids. All peristaltic pumps were calibrated prior to the start of the experiment. The CM-1 overhead paddles (Muscle Corp., Osaka, Japan) stirred at a rate of 20 rpm in the gastric and duodenal chambers. For every 25 s, a high-speed, quick burst (500 rpm) was cyclically repeated to mimic gastrointestinal (GI) contractions and to homogenize the compartment facilitating the solid particle transfer from one chamber to the next one. The jejunal chamber was stirred with a magnetic bar at an approximate rate of 50 rpm. All experiments were performed at 37 °C. After 60 min, pumps were shut down as the gastric content was emptied. Concentrations in the GIS_duodenum_ and GIS_jejunum_ were still measured up to 120 min. Samples were withdrawn from the GIS compartments at predetermined time-points up to 120 min in order to measure the dissolved amount of DKT. The pumps and overhead paddles were controlled by an in-house computer software program. Solution concentrations were determined by centrifuging 300 µL of the withdrawn sample for 1 min at a speed of 17,000 *g* (AccuSpin Micro 17, Fisher Scientific, Pittsburgh, PA, USA). After centrifugation, 100 µL of the supernatant was diluted 1:1 with MeOH, and the MeOH sample was diluted 1:1 again with 0.1 N HCl and transferred to high performance liquid chromatography (HPLC) capped vials. All obtained samples were analyzed by HPLC (see below Section 2.8).

### 2.3. Design of the In Vitro Dissolution Studies Performed with USP-2

To investigate the impact of each region of the human GI tract separately, single-compartmental dissolution studies were performed. Dissolution studies in the USP-2 (paddle) apparatus were performed at 37 °C and 30 rpm in 500 mL of fluid. Three tablets of each formulation were tested in four different media: (1) FaSSIF-v1 at pH 6.5; (2) 0.01 N HCl (pH 2); (3) 0.01 N HCl + 34 mM NaCl; and (4) 0.01 N HCl + 135 mM. The concentrations of Na^+^ and Cl^−^ measured in human gastric fluids are equal to 68 ± 29 mM and 102 ± 28 mM, respectively [6]. Samples of 500 µL were taken and immediately centrifuged and diluted as described previously.

### 2.4. In Silico Deconvolution to Obtain In Vivo Bioavailability Input Rate

Intravenous pharmacokinetic data were obtained from Valles and co-workers [7]. A two-compartmental pharmacokinetic (PK) open model was fitted to the data to get DKT disposition constants as depicted in Table 4.

PK parameters were used to apply Loo–Riegelman mass balance deconvolution method in order to obtain the plots of bioavailable fractions versus time profile of all the assayed formulations. As oral plasma data were obtained from different BE studies, the plasma concentration–time profiles for all test formulations were normalized using the reference formulations ratios at each time point between both BE studies [8,9]. Similar normalization results were obtained by using the area under the curve (AUC) references ratios (data not shown).

### 2.5. Description of the Two-Step In Vitro–In Vivo Correlation (IVIVC)

Fractions dissolved in jejunal chambers of each formulation were used to develop the two-step IVIVC. To estimate the fractions dissolved, the maximum amount of DKT dissolved among the three formulations was used to transform amounts into fractions. Bioavailable fractions obtained by Loo–Riegelman method of each formulation at each time point versus the fractions dissolved of the corresponding formulation at the same time points were represented. For non-coincident sampling times in vitro versus in vivo, the corresponding dissolved or absorbed fractions were estimated by linear interpolation between the previous and next time point. The obtained IVIVC relationship was internally validated—theoretical fractions absorbed were calculated from the experimental fractions dissolved by using the IVIVC equation. The fractions absorbed were back-transformed toward concentrations by applying Equation (1) [10].

(1) CT=(XA)TVc− (XP)T−1Vc e−K21Δt+ CT−1K12Δt2− CT−1K12K211−e−K21Δt−CT−1KelΔt2−AUCT−1Kel1+K12Δt2+KelΔt2

where *C_T_* is the plasma concentration at time *t*; *C_T−_*_1_ is the plasma concentration at the previous time point (*T −* 1); (*X_A_*)*_T_* is the absorbed amount at time *t*; (*X_P_*)*_T−_*_1_ is the amount in the peripheral compartment at the previous sampling time; Δ*t* is the time interval between two consecutive sampling times; *V_c_* is the central compartment volume; *K*_12_ and *K*_21_ are the distribution constants and *K_el_* the elimination rate constant from the central compartment. The peripheral “concentrations” were estimated with Equation (2) [11]:

(2) $$P_{t}=K_{12}\cdot e^{-K_{21}\cdot t}\cdot \int_{0}^{t}C\cdot e^{K_{21}\cdot t}\partial t$$

where *K*_12_ and *K*_21_ are the values obtained previously from literature in Table 4.

The predicted plasma levels were used to estimate plasma *C*_max_ and AUC predicted values to be compared with the experimental ones and to estimate the relative prediction error (Equation (3)):

(3) $$RE\%=100\times \left(\frac{experimental\;value-predicted\;value}{experimental\;value}\right)$$

### 2.6. Evaluation of DKT to Free Acid Conversion Pathways/Kinetics During Salt Dissolution

DKT to free acid conversion was studied in situ by optical microscopy. The studies were conducted at room temperature (22–23 °C) using an inverted optical microscope (Leica DMi8, Wetzlar, Germany) and 10×, 20×, or 40× magnification objective lenses. An inverted microscope has the advantage of a long focal length that allows examination of the phases formed during dissolution without having to remove the solution. Two concentration levels of both DKT and excipients were studied by varying the amount of DKT and excipients added to 96-well plates followed by the addition of 300 µL of hydrochloric acid (pH 2 (0.01 M) and 34.2 mM NaCl) with pre-dissolved tablet excipients. The influence of excipients was determined by dissolving formulation excipients in the dissolution media prior to DKT salt addition. The high concentration level (C_H_) corresponds to 685 ± 23 µg of salt added to a 300 µL aliquot of a solution of 1 tablet dissolved in 20 mL, whereas the low concentration (C_L_) corresponds to 38 ± 1 µg of salt added to a 300 µL aliquot of 1 tablet dissolved in 300 mL. From that point of view, the high concentration (C_H_) is 18 times higher than the low concentration (C_L_).

Brightfield images were collected with a Leica DMC2900 camera controlled with LAS v4.7 software (Leica Microsystems, Wetzlar, Germany). Solid particles of the free acid were added at two different levels, representing <3% (*w/w*) and 3% (*w/w*) relative to the total amount of salt present in the well. In that way, the influence of salt purity on drug precipitation could be determined.

### 2.7. Solubility and pH_max_ Determination

Drug solubility was measured by adding the DKT to solutions at various pH values and stirring at 37 °C for 24 h. The pH was adjusted by adding HCl or NaOH to the solutions. Solubility values were used to calculate the salt solubility product, *K_sp_*, according to the following equation

(4) $$K_{sp}=\left[DK^{-}\right]\left[TH^{+}\right]$$

where [*DK*^−^] represent the concentration of ionized drug and [*TH*^+^] represents the concentration of counterion. The pH_max_ was calculated from the intersection of the DKT and free acid solubility curves generated according to equations presented in the results section. The pH_max_ refers to the pH where both the DKT and free acid have equal solubilities.

### 2.8. Concentration Analysis of DKT by HPLC

DKT concentrations in the samples were measured by HPLC-UV (Hewlett Packard series 1100 HPLC Pump combined with Agilent Technologies 1200 Series Autosampler). A volume of 75 µL was injected into the HPLC system (Waters 515 HPLC Pump with Waters 717 Autosampler). DKT was detected with an UV lamp at 262 nm (Water 996 Photodiode Array Detector). The mobile phase consisted of 60:40 mixture of acetonitrile and purified water A (both containing 0.1% TFA). Stationary phase was a C-18 Agilent Eclipse XDB (4.6 × 150 mm; 3.5 µm). Elution flow was 1 mL/min and retention time for DKT was 3.95 min. Calibration curves were made in mobile phase based on a stock solution of DKT in methanol. Linearity was observed between 1.5 µg/mL and 300 µg/mL covering all the experimental sample values. The observed peaks were integrated using Millenium software (Agilent Technologies, County of Santa Clara, CA, USA). The developed analytical method met the standards for precision and accuracy.

### 2.9. Data Analysis and Presentation

Dissolution profiles of DKT in all GIS compartments were plotted either as drug concentration or mass of drug versus time (average ± standard deviation; n = 4). Dissolution profiles from USP-2 experiments were represented as the fraction dose dissolved versus time (average n = 3).

## 3. Results and Discussion

### 3.1. Solubilities and Solution Stabilities of DKT and Free Acid Solid Forms as a Function of pH

Dexketoprofen is a lipophilic (LogP 3.61) weak acid with pK_a_ of 4.02 at 37 °C [12,13]. The DKT salt was developed to enhance its solubility over the free acid and improve dissolution in the GI tract. Salt formation is a well-known strategy to increase the solubility of either lipophilic weak acids or bases in order to improve oral absorption. Nevertheless, the expected benefits of forming a salt may not work if the level of supersaturation leads to drug precipitation to the free acid or base, thereby reducing the drug exposure levels for absorption [14,15,16]. The “supersaturation/precipitation interactive process” depends on the characteristics of the weak acid or base and, not unimportant, on the dissolution study design with respect to media composition and hydrodynamics that will determine the bulk and interfacial pH around the dissolving particles.

**Figure 2:**
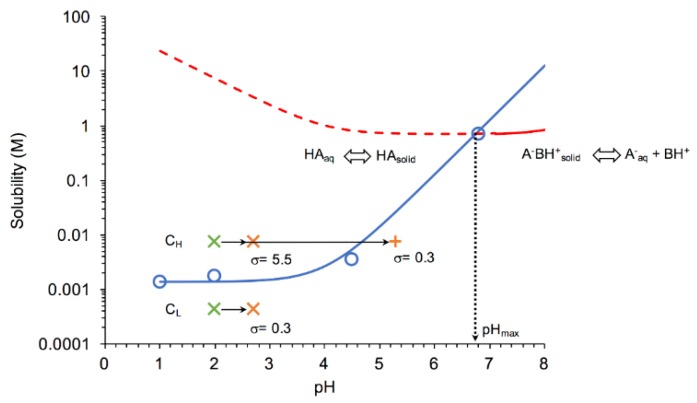
Solubility−pH dependence of free acid and DKT salt indicating the stability regions for salt and free acid solid-state forms and the conditions under which dissolution-precipitation microscopy studies were carried out. Salt has a pH_max_ of 6.7 below which supersaturation with respect to free acid can occur. Two salt concentrations were studied: C_L_ represents the low dose concentration. C_H_ represents a higher concentration of 18x C_L_, as described in the Materials and Methods section. Arrows represent the pH changes that different salt formulations experienced. Green X represents initial concentration and pH. As the salt dissolves, the bulk pH increased to 2.7 ± 0.2 (orange X) for the bioequivalence (BE) and reference formulation excipients, whereas the pH increased up to 5.3 (orange+) for the non-bioequivalence (non-BE) formulation excipients. Solubility curve for salt (red line) was calculated from Equation (6), using *K*_sp_ and pK_a,DK_ reported in the text and pK_a,T_ = 8.1 [17]. The free acid solubility curve (blue line) was calculated according to Equation (7). Open circles represent DK measured solubilities at 37 °C. The dashed red line represents supersaturated conditions with respect to DK if solutions are saturated with salt.

The influence of pH on the stability of the DKT salt and DK free acid was determined by examining the solubility-pH profiles presented in Figure 2. These results show that DKT salt has a pH_max_ at 6.7, where both salt and free acid have equal solubilities; thus, both phases are stable. Below pH_max_, the salt is more soluble than the free acid, and generates supersaturation with respect to the acid. Supersaturation is expressed as the ratio of salt to free acid solubility, S_salt_/S_acid/intrinsic_. The lower the pH below pH_max_, the higher is the supersaturation that the salt may generate, and the higher is the driving force for salt to free acid conversion. On the other hand, the salt is stable at pH ≥ pH_max_.

Given the salt solubility at pH_max_ and the free acid S_0_ values, supersaturation with respect to free acid can be very high (>500) causing drug precipitation and depletion of drug concentration levels. Salt to drug conversions were examined by microscopy at two salt concentrations, one equivalent to the dose and one higher, as indicated in the graph. Although at C_L_ the bulk solution is undersaturated with respect to free acid, the salt particles can exhibit supersaturation at the salt/liquid interface as this region is saturated with respect to salt. The solubility−pH profiles for the salt and the free acid were generated according to equations derived from the solution chemistry equilibria. For the salt, the equilibrium reaction is

(5) $$DK^{-}TH^{+}\overset{K_{sp}}{↔}DK^{-}+TH^{+}$$

The equilibrium constant for this reaction is the salt solubility product (*K*_sp_) given by Equation (4). *K_sp_* of DKT was determined to be 4.96 × 10^−1^ M^2^, from the measured [*DK^−^*] or salt solubility, S_salt_ = 7.04 × 10^−1^ M at pH 6.8. While *K*_sp_ is constant with pH, salt solubility is not, and its dependence on pH is given (assuming no precipitation of protonated dexketoprofen and no solubility-limiting effect by other ions in the medium) by

(6) $$S_{salt}=\sqrt{K_{sp}\left(1+10^{pK_{a,DK}-pH}\right)\left(1+10^{pH-pK_{a,T}}\right)}$$

where *K_a,DK_* and *K_a,T_* are the acid and base dissociation constants of the salt constituents. For the free acid, the solubility in terms of pH is expressed by

(7) $$S_{acid}=S_{0}\left(1+10^{pH-pK_{a,DK}}\right)$$

where *S*_0_ is the intrinsic solubility of the free acid, determined to be 1.36 × 10^−3^ M. The pH_max_ was also calculated applying the following equation:

(8) $$pH_{max}=pK_{a,DK}+log\frac{\sqrt{K_{sp}}}{S_{0}}$$

obtained by solving Equations (6) and (7) for pH when S_salt_ = S_acid_ at pK_a,DT_ < pH < pK_a,T_, under conditions where both drug and counterion are fully ionized. The pH_max_ value of 6.7 obtained by this equation is equal to that obtained graphically because trometamol is still almost completely ionized at this pH.

### 3.2. Formulation Performance of the DKT Formulations in the GIS with Protocols 1 and 2

**Figure 3:**
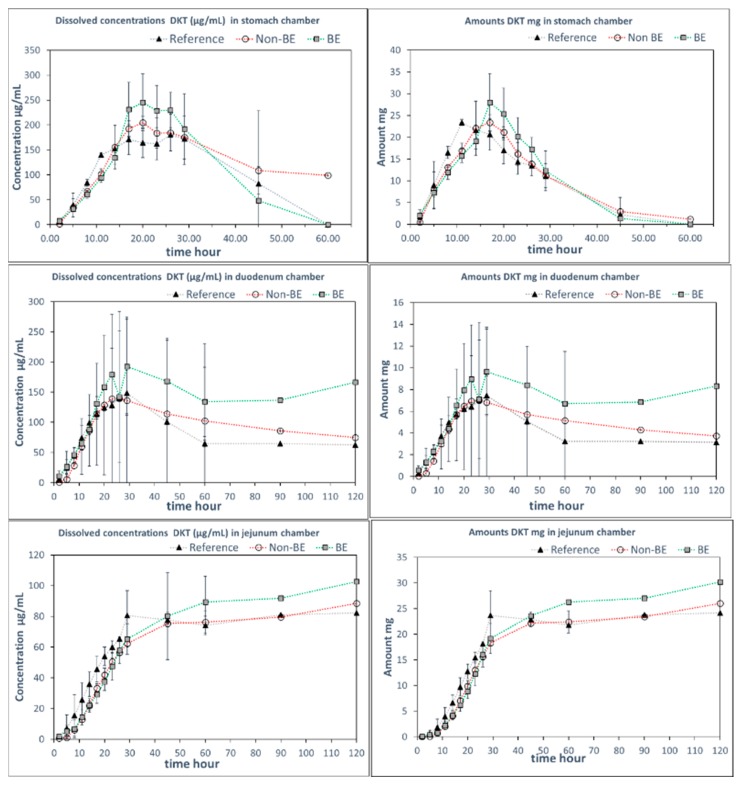
DKT concentrations (**left panels**) and amounts in solution (**right panels**) in the GIS_stomach_, GIS_duodenum_, and GIS_jejunum_ vessels obtained with Protocol 1 with HCl 0.01M in GIS_stomach_ and phosphate buffer 50 mM (pH 6.8) in the duodenal chamber (n = 3). Standard deviations overlap over the three profiles and they are not shown. Dotted lines are included to facilitate visual profile comparison.

**Figure 4:**
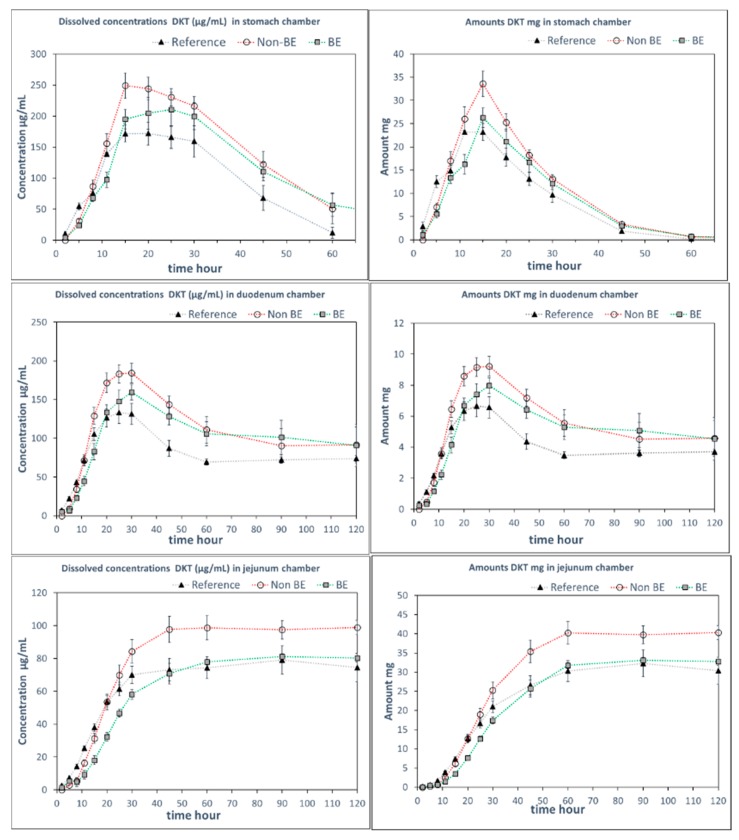
DKT concentrations (**left panels**) and amounts in solution (**right panels**) in the GIS_stomach_, GIS_duodenum_, and GIS_jejunum_ vessels obtained with Protocol 2 with NaCl in GIS_stomach_ at 34 mM and FaSSIF-v1 in the duodenal chamber. Experimental data were shown as mean ± SD (n = 4). Dotted lines are included to facilitate visual profile comparison.

Since the GIS can incorporate the dynamic shift in fluid pH and composition as the dosage form transits from the stomach to the intestine, it has previously shown utility in predicting the in vivo performance of weak bases [4,18,19,20,21,22]. In this study, GIS dissolution experiments were performed using two different protocols, representing two different medium compositions in the gastric and intestinal compartments. Whereas Protocol 1 contained SGF in the gastric compartment and pH 6.8 phosphate buffer in the intestinal compartments, in Protocol 2, sodium chloride was added to SGF in the gastric compartment and FaSSIF-v1 was added to the intestinal compartment. Figure 3 and Figure 4 include the observed solution DKT concentrations as a function of time for the three different formulations as tested in the GIS device, applying Protocol 1 and Protocol 2, respectively.

Remarkably, differences in dissolution behavior were observed in the gastric compartment of the GIS apparatus in the presence and absence of NaCl. When NaCl was absent from the gastric medium (Protocol 1), the gastric dissolution profiles did not discriminate between the three formulations. However, when NaCl was added to the gastric medium (Protocol 2), differentiation was observed between the formulations, whereby the non-BE formulation dissolved earlier and to a great extent, as was observed in vivo (deconvoluted profiles). The addition of NaCl to SGF resulted in observed differences in the disintegration behavior in the gastric chamber as discussed further in the next section.

**Figure 5:**
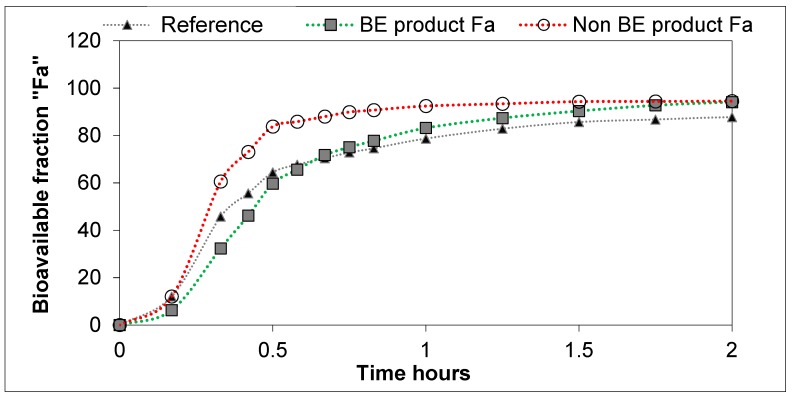
Bioavailable DKT fractions obtained from plasma levels through Loo–Riegelman deconvolution.

The differences in dissolution rates across the three formulations as observed in the GIS_stomach_ with Protocol 2 were maintained after the transfer to the duodenal chamber. Finally, the GIS_jejunum_ accumulated the differences and the jejunum cumulative dissolution profiles of the three assayed formulations followed the same trend as the oral fractions absorbed obtained from deconvolution of plasma profiles, as depicted in Figure 5.

Measured DKT concentrations in the GIS_stomach_ were the result of the balance between the supersaturation factor of DKT promoted by the salt and the precipitation of the free acid. That balance evolved differently in the presence or in the absence of NaCl. Potential reasons for these observations are the differences in solubility of sodium dexketoprofen versus the trometamol salt and the increased solubility of calcium monohydrogen phosphate in the presence of NaCl. After transfer to the duodenal chamber, a reflection of the gastric dissolution profiles was observed in the FaSSIF-v1 media, using Protocol 2. In Protocol 1, no differences between dissolution profiles were observed in the duodenal chamber. It could be due to the fact that the three formulations already behaved similarly in the GIS_stomach_ but, on the other hand, the higher buffer strength of the 50 mM phosphate buffer used in Protocol 1 readily promoted DKT dissolution hiding the effect of calcium phosphate on solid surface pH.

**Figure 6:**
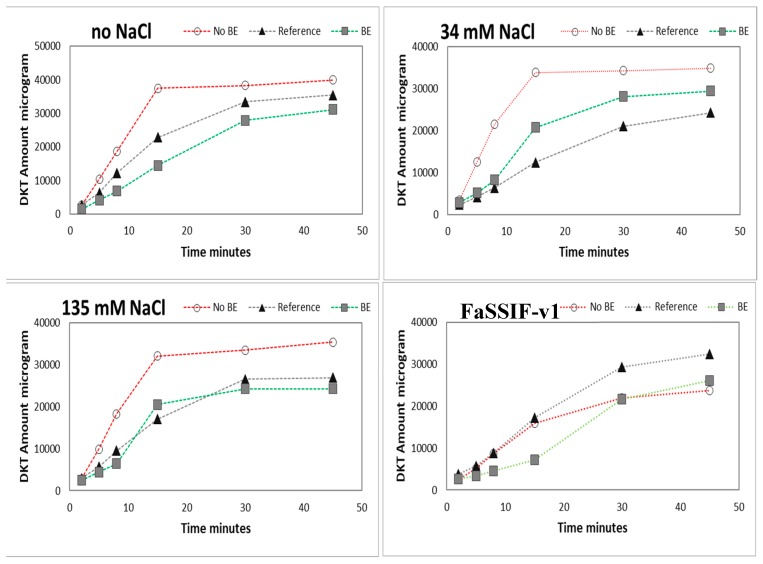
Amounts of DKT in micrograms dissolved in USP-2 apparatus (500 mL; 50 rpm) in acidic media (HCl 0.01M pH = 2) at different levels of NaCl content and in FaSSIF-v1 media. Data are presented as means (*n* = 3).

As for why did the differences between the studied formulations appear with Protocol 2 but not Protocol 1, the USP paddle dissolution results in FaSSIF-v1, shown in Figure 6, indicate that the incorporation of NaCl into the simulated gastric fluid rather than the use of FaSSIF-v1 is the primary reason. This is rather intriguing due to the low NaCl molarity present in the GIS_stomach_ owing to the six-fold dilution with water. Applying the ionic strength and activity coefficient calculations shows that any effect on the DKT and/or calcium phosphate behavior would be marginal at those NaCl concentrations. Our current hypotheses for possible causes have not yet been experimentally tested. Therefore, additional future studies are planned to investigate the possible causes behind this effect.

### 3.3. The Impact of NaCl on Disintegration and Dissolution

Results of the dissolution experiments in the USP-2 apparatus using four different media (FaSSIF-v1 at pH 6.5; 0.01 N HCl (pH 2); 0.01 N HCl + 34 mM NaCl; and 0.01 N HCl + 135 mM), which were designed to explore the impact of NaCl on formulation disintegration/drug dissolution, are depicted in Figure 6. The different concentrations of NaCl cover the observed values as observed in the human stomach [6].

The presence of NaCl mainly affected the disintegration and dissolution process of the non-BE formulation resulting in an enhanced dissolution rate in the presence of NaCl, which was not observed for the BE-formulation and the reference drug product. While performing these dissolution experiments, remarkable differences in disintegration behavior could also be observed between the non-BE formulations and the other two formulations.

The faster dissolution of DKT from the non-BE formulation compared to the other formulations under Protocol 2 is most likely related to the high content of calcium phosphate in the tablet core of the non-BE formulation, which is not present in the other formulations. This high level of calcium phosphate in the tablet core of the non-BE formulation can increase the pH at the solid surface accelerating the dissolution of DKT and also facilitating tablet disintegration. Modulation of microenvironmental pH has been shown as an effective strategy to modulate the dissolution rate of GDC-0810, a weak acid of an oral anticancer drug, by using sodium bicarbonate to change solid surface pH [23]. This same strategy of using pH-modifiers has been proposed as a release modulating mechanism in solid dispersions [24] and other immediate-release dosage forms [25]. Solid surface pH data was not obtained in these dissolution experiments and bulk pH values of the media during dissolution experiments were available only in GIS_stomach_ at 13 min with Protocol 2. At that time, the non-BE formulation containing calcium phosphate presented a pH of 3.5, 1 unit higher than the pH of the reference and BE formulation that was approximately 2.5. Calcium phosphate can increase the pH at the solid surface of the drug-excipients particles, then increasing DKT solubility, and consequently decreasing the degree of supersaturation, which will, subsequently, prevent or reduce the precipitation gradient [25]. Besides calcium phosphate, FaSSIF-v1 surfactants seem to play a major role in the supersaturation/free acid precipitation balance as it has been reported for other ionizable compounds [26,27,28].

### 3.4. In Vitro–In Vivo Correlations (IVIVC) for the Different Drug Products

**Figure 7:**
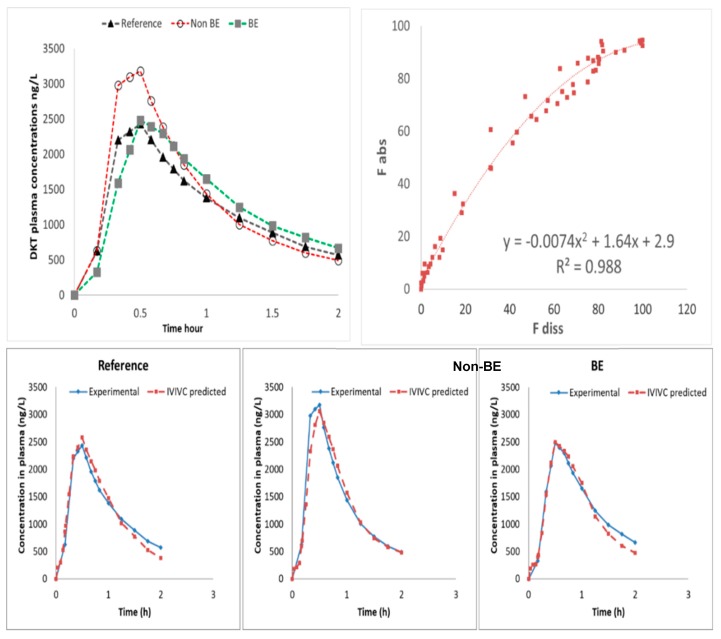
Invitro–invivo correlation (IVIVC) level A. (**Top left plot**) Human plasma levels of DKT after oral administration of the reference product (Enantyum^®^) and two test formulations from two BE trials. Concentrations of test products were normalized using the ratio of reference concentrations. Plasma levels are shown up to 2 h to highlight C_max_ differences. (**Top right plot**) Non-linear IVIVC plotting fraction absorbed versus fraction dissolved. (**Bottom plots**) Internal validation through prediction of plasma levels for each formulation.

When fractions absorbed of the three formulations were plotted against the fractions dissolved in jejunal chamber when Protocol 2 was applied, a single relationship was obtained, indicating dissolution was the limiting factor for DKT systemic input (Figure 7).

Nevertheless, the obtained relationship is not linear but curved due to a time-scale shift from in vivo to in vitro. In vivo dissolution and, consequently, absorption is faster than what was simulated in vitro. A time-scaling approach was not considered to be necessary as the time shift was less than 30 min and the non-linear equation presented a good predictive performance. The reason for the slight time shift could be the fact that jejunal dissolved amounts were used while in vivo dissolution/ absorption from duodenum can play a relevant/significant role. The internal validation of the obtained IVIVC was done by estimating fractions absorbed from the experimental dissolved ones using the obtained non-linear equation and then back-transforming fractions absorbed in plasma levels. Relative prediction errors of plasma C_max_ and AUC were lower than 10% for all the formulations (Table 5).

### 3.5. Differences between Drug Salt to Free Acid Conversions for BE and non-BE Formulations

**Figure 8:**
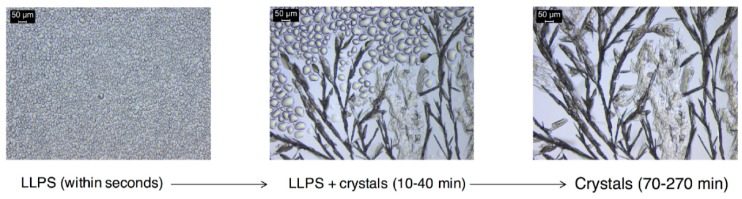
Diagram showing the DKT transformations to DK drug phases during dissolution. A more soluble drug phase appears first as liquid–liquid phase separation (LLPS), followed by a less soluble crystalline phase. The kinetics and pathways of these transformations are dependent on salt concentration, formulation excipients and level of drug phase impurity in the salt phase.

Drug exposure levels are influenced by the kinetics of salt dissolution and drug precipitation as well as the evolution of drug phases. Microscopic examination of salt dissolution in pH 2 identified two main pathways depending on the formulation excipients: (1) salt dissolved without conversion in the presence of calcium phosphate as one of the excipients (non-BE formulation), (2) salt dissolution formed a nano-phase that grew to spherical and island morphologies that converted to free acid crystals (Figure 8). The time course of the second pathway was dependent on the salt/excipient concentration and the presence of free acid crystals in the salt phase.

Shown in Figure 8 is the precipitated phase that appears as non-coalescing drops suspended in the dissolution media. This phase surrounds the fast dissolving salt particles, within seconds. Conversion of this fine precipitate to drug crystals was observed after 2 min or longer (up to 1 h) depending on initial salt concentration, formulation excipients, and presence of drug impurity in salt.

The massive phase separation appears initially hazy as its size is in the submicron range and below the level of detection of the microscope. This phenomenon has been referred to as spinodal, oiling out, or liquid–liquid phase separation (LLPS), consistent with that observed for other weakly basic drugs under high supersaturations, such as ritonavir [29,30,31,32,33]. The supersaturations with respect to drug, generated at the surface of the dissolving DKT salt particles are very high (>500 at pH 2) based on the solubilities of the salt and drug forms shown in Figure 2. While the interfacial pH was not evaluated, the surface of the dissolving salt is saturated with respect to the salt and generates much higher supersaturations than those in the bulk dissolution media. In fact, the appearance of LLPS occurred even when the bulk solution was below the drug solubility (σ = 0.3) (Figure 2).

Table 6 summarizes DKT transformations to drug phases during dissolution. Observed dissolution of all phases initially or after the appearance of LLPS and drug crystals is consistent with undersaturated drug conditions in the bulk solution at dose concentration (C_L_). Non-BE formulation excipients in the media at higher concentrations (C_H_) increased pH to 5.3, and no precipitation was observed as the solution concentration is below salt and drug solubility. This is most probably because of the neutralization of HCl by the large level of calcium phosphate present. REF and BE formulation excipients exhibited different conversion behavior at C_H_; LLPS formed and crystallized after 10–30 min. The presence of drug crystals as impurity (less than or equal to 3%) in the salt phase led to the faster conversion of LLPS to less soluble drug crystals, i.e., faster drug crystallization and LLPS dissolution. Faster conversion rates result in lower drug exposure levels.

It is important to consider that concentration levels varied for both salt and excipients, as the excipient concentrations were varied by diluting the dissolved tablet prior to adding the salt. Therefore, the different behavior of the high concentration of excipients with the non-BE shows the key role of alkalinizing excipients on stabilizing the salt, as the pH approaches the pH_max_.

Furthermore, the dilution of the gastric fluid by water in the GIS_stomach_ of the GIS setup explains the C_H_ results better matching the trend of the GIS data than the C_L_ results. This is because the lower HCl concentrations caused by dilution were not sufficient to eliminate the pH differences caused by the presence of calcium phosphate in the non-BE formulation. This gave rise to an end effect similar to the high calcium phosphate levels under C_H_ conditions being able to effectively neutralize the 0.01 M HCl. This is supported by the aforementioned observation of higher pH value in the GIS_stomach_ for the non-BE formulation compared to the reference one, which is more in line with the C_H_ than with the C_L_ results.

## 4. Conclusions and Future Directions

Differences in dissolution behavior between the BE and non-BE DKT products are a result of drug salt to free acid phase conversion rates and mechanisms. In this case, the presence of an alkalinizing excipient in a tablet formulation of a salt of a weakly acidic drug suppresses salt disproportionation as pH approaches pH_max_, leading to a higher extent of drug dissolved and failing BE requirements. This is a case where salt disproportionation appears to modulate the behavior of a highly soluble salt in a favorable way by the formation of a transient phase prior to crystallization of the less soluble free acid. Rates of formation of less-soluble drug phases, LLPS, and crystal forms during DKT salt dissolution are dependent on the excipients, dissolution pH, and presence of DK free acid as an impurity in the salt. Excipients that increase pH (calcium phosphate) decreased free acid precipitation and enhanced dissolved levels of drug in the non-BE formulation. The BE product was associated with a faster conversion to KT crystals, whereas non-BE product experienced less drug precipitation under the same condition. Generic and non-generic DKT formulations were discriminated in vitro in the GIS device by adding NaCl to SGF and using FaSSIF-v1 media in the duodenum compartment. However, the relevant GI variables for the development of “In Vivo Product Predictive Dissolution Methods” need to be adapted to each compound. The selection of particular dissolution conditions as media and secretion fluids composition for the GIS device will depend on (i) the BCS profile of the drug, (ii) its ionization characteristics, and (iii) its formulation characteristics (e.g., presence of calcium monohydrogenphosphate). The ionic strength impact as well as the surfactants effects on the supersaturation/precipitation balance needs to be further investigated.

## Figures and Tables

**Table 1:** Qualitative differential composition of the reference marketed drug product and the test products. The ingredients in bold are the added excipients to the core or the coating of the tablet for both test products, which was not presented in the reference marketed drug product.

Dexketoprofen 25 mg (as Dexketoprofen Trometamol 36.9 mg) Film-Coated tablets	Qualitative Composition of Excipients
Reference marketed drug product (Enantyum^®^)	Core: microcrystalline cellulose, maize starch, glycerol distearate, sodium starch glycolate Coating: hypromellose, titanium dioxide, polyethylene glycol (PEG) 600, and propylene glycol
Test product (bioequivalence (BE))	Core: microcrystalline cellulose, maize starch, glycerol distearate, sodium starch glycolate, magnesium stearate and colloidal silica * Coating: hypromellose, titanium dioxide, polyethylene glycol (PEG) 600, propylene glycol, **macrogol 6000 and talc**
Test product failing BE study (Non-BE)	Core: microcrystalline cellulose, maize starch, glycerol distearate, sodium starch glycolate, **magnesium stearate, colloidal silica and calcium monohydrogen phosphate** Coating: hypromellose, titanium dioxide, polyethylene glycol (PEG) 600, and propylene glycol, **macrogol 6000 and talc**

* The ingredients that listed in bold in Table 1 represent the differences between the test and reference products. These excipients were not included in the marketed reference product.

**Table 2:** Overview of dissolution media, initial volumes, and secretion rates applied in the gastrointestinal simulator (GIS) device for the first set of standard dissolution experiments (i.e., standard dissolution settings). The jejunal compartment was empty at the start of the experiment.

Fasted State Test Condition Protocol 1	GIS_stomach_	GIS_duodenum_
Dissolution media	Simulated gastric fluid (SGF), pH 2.0, 0.01 M HCl	Phosphate buffer, pH 6.8–50 mM
Initial volume	50 mL SGF + 250 mL of tap water	50 mL
Secretions	1 mL/min of SGF	1 mL/min of phosphate buffer, pH 6.8–100 mM

**Table 3:** Overview of dissolution media, initial volumes, and secretion rates applied in the GIS device for the second set of dissolution experiments with a higher level of biorelevance by adding NaCl to SGF and by adding sodium taurocholate and lecithin to the phosphate buffer in order to obtain fasted state simulated intestinal fluids (FaSSIF-v1). The jejunal compartment was empty at the start of the experiment.

Fasted State Test Condition Protocol 2	GIS_stomach_	GIS_duodenum_
Dissolution media	Simulated gastric fluid (SGF), pH 2.0, 0.01 M HCl + 34.2 mM NaCl	FaSSIF-v1 (pH 6.5)
Initial volume	50 mL SGF + 250 mL of tap water	50 mL
Secretions	1 mL/min of SGF	1 mL/min of 4 times concentrated FaSSIF-v1 (4x FaSSIF-v1)

**Table 4:** Disposition parameters of DKT for a two-compartmental pharmacokinetic (PK) model: V1 represents the central compartment volume; *K*_10_ represents a first-order elimination rate constant; *K*_12_ and *K*_21_ reflect the two rate constants distributing the drug between the peripheral and central compartment, respectively.

Parameter	Unit	Value	Standard Error	CV%
*V*_1_	mL	3549.53	201.23	5.67
*K*_10_	1/h	1.64	0.08	5.14
*K*_12_	1/h	0.93	0.13	14.34
*K*_21_	1/h	0.96	0.09	8.91

**Table 5:** Experimental and predicted plasma *C*_max_ and area under the curve (AUC) values for all DKT formulations and relative prediction errors.

	*C*_max_ Exp ng/L	*C*_max_ Pred ng/L	RE%	AUC Exp ng/L*h	AUC Pred ng/L*h	RE%
**Reference**	2430.6	2576.3	–5.99	2497.3	2520	–0.92
**Non-BE**	3177.3	3062.5	3.61	2785.7	2739	1.69
**BE**	2478.5	2491.9	–0.54	2626	2538	3.35
**Average**			3.38			1.98

**Table 6:** Evolution of drug phases during DKT dissolution in different formulation conditions.

Formulation	Condition	LLPS	Crystal	Full Dissolution	Final pH
Non-BE	C_H_^b^	–	–	+ ^a^	5.3
	C_L_^c^	+	–	+(80 min)	2.8
	C_L_ + DK solid ^d^	+	+	+(>7 h)	2.9
BE	C_H_	++	++	NA	2.9
	C_L_	+	–	+(90 min)	2.6
	C_L_ + DK solid	+	+	+(>4 h)	2.8
REF	C_H_	++	++	NA	2.9
	C_L_	+	–	+(60 min)	2.6
	C_L_ + DK solid	+	+	+(>2 h)	2.7
Salt in buffer no excipients	C_H_	++	++	NA	2.7
	C_L_	+	–	+(85 min)	2.7
	C_H_ + DK solid	++	++	–	2.7

^a^ within seconds; ^b^ C_L_, low concentration, dose in 300 mL solution (total drug concentration = 4.3 × 10^−4^ M); ^c^ C_H_, high concentration = 18 × C_L_ (total drug concentration = 7.7 × 10^−3^ M); ^d^ DK solid, represents less than or equal to 3% free acid drug as impurity in the salt phase; and NA, not applicable as C_H_ at this final pH is above free acid solubility.

## Abbreviation

DKT	dexketoprofen trometamol
DK	dexketoprofen
FaSSIF-v1	fasted state simulated intestinal fluids version 1
SGF	simulated gastric fluids
HPLC	high-performance liquid chromatography
LLPS	liquid–liquid phase separation
PK	pharmacokinetics
GIS	gastrointestinal simulator
USP	United States Pharmacopeia
GI	gastrointestinal
BE	bioequivalence
AUC	area under the curve
C_max_	maximal concentration
C_H_	high concentration of DKT
C_L_	low concentration of DKT
IVIVC	in vitro–in vivo correlation
REF	reference listed drug product
Non-BE	non-bioequivalent
S_salt_	Solubility of the salt form of dexketoprofen (i.e., dexketoprofen trometamol)
S_acid_	Solubility of the salt form of dexketoprofen
NaCl	sodium chloride
S_0_	intrinsic solubility of dexketoprofen
pH_max_	the pH_max_ refers to the pH where both the DKT and free acid have equal solubilities.
K_sp_	salt solubility product
TH^+^	positively ionized trometamol
GIS_stomach_	gastrointestinal simulator gastric chamber
GIS_duodenum_	gastrointestinal simulator duodenal chamber
GIS_jejunum_	gastrointestinal simulator jejunal chamber
F_a_	fraction absorbed
HA	acid form
A^−^	negative ionized acid form
BH	protonated basic form
RE%	relative prediction error in percentage
EMA	European Medicines Agency
BCS	biopharmaceutics classification system
NaOH	sodium hydroxide
HCl	hydrochloric acid
TFA	trifluoracetic acid
